# Effects of plaza dancing on body composition and cardiopulmonary function in middle-aged and the aged healthy women: a systematic review and meta-analysis

**DOI:** 10.3389/fpubh.2025.1667818

**Published:** 2025-09-08

**Authors:** Jiayi Yao, Haozhe Wang, Shiguan Jia, Mingyu Liao, Wenjia Chen

**Affiliations:** ^1^School of Physical Education, China University of Mining and Technology, Xuzhou, China; ^2^Aviation Safety and Security College, Civil Aviation Flight University of China, Deyang, China

**Keywords:** plaza dancing, middle-aged and the aged women, body composition, cardiorespiratory fitness, meta-analysis

## Abstract

**Background/objectives:**

Middle-aged and older women aged 45 and above face problems of body composition imbalance and declining cardiopulmonary function due to physiological changes during menopause, while traditional exercise interventions have adaptability deficiencies. Plaza dancing, as a form of collective aerobic dance exercise performed to music in open spaces such as plazas and parks, is characterized by low intensity and ease of learning, and has demonstrated health promotion potential. However, there is a lack of systematic evaluation of its comprehensive effects on body composition and cardiovascular function in middle-aged and older women. This study aims to clarify the effectiveness of plaza dancing on body composition and cardiovascular function in healthy middle-aged and older women through systematic review and meta-analysis, and to explore the dose–response relationship of intervention duration.

**Methods:**

Following the PRISMA guidelines and Cochrane Collaboration Handbook, we systematically searched PubMed, Web of Science, CNKI, Wanfang, and VIP databases (up to July 5, 2025), and included 38 studies meeting the criteria, with 17 studies entering meta-analysis. The Cochrane Risk of Bias tool (ROB 2.0) was used to assess study quality. Statistical analyses were performed using Review Manager 5.4 and R 4.5.1 to calculate standardized mean differences (SMD) or mean differences (MD). Random-effects models (I^2^ > 50%) or fixed-effects models (I^2^ < 50%) were applied based on heterogeneity, with subgroup analyses and publication bias assessments conducted.

**Results:**

Meta-analysis revealed that plaza dancing significantly reduced body weight (SMD = −0.27, 95%CI [−0.46, −0.09], *p* = 0.004), BMI (SMD = −0.59, 95%CI [−0.85, −0.33], *p* < 0.00001), body fat percentage (SMD = −0.51, 95%CI [−0.82, −0.20], *p* = 0.001), resting heart rate (SMD = −0.38, 95%CI [−0.68, −0.07], *p* = 0.02), systolic blood pressure (SMD = −0.42, 95%CI [−0.76, −0.09], *p* = 0.01), cholesterol (MD = −0.25, 95%CI [−0.48, −0.02], *p* = 0.03), and triglycerides (MD = −0.20, 95%CI [−0.34, −0.06], *p* = 0.005), while significantly improving vital capacity (SMD = 0.76, 95%CI [0.25, 1.26], *p* < 0.0001). Subgroup analysis indicated that BMI improvement was more significant when intervention duration ≤12 weeks (SMD = −0.69). Publication bias assessment showed that results for most indicators were robust. Meta-regression analysis revealed significant dose–response relationships: both BMI (*β* = 0.043, 95%CI [0.025, 0.061], *p* < 0.001, R^2^ = 0.998) and resting heart rate (β = 0.041, 95%CI [0.010, 0.072], *p* = 0.011) demonstrated significant negative time effects, with greater effect sizes observed in short-term interventions (8–12 weeks). Triglycerides exhibit a unique “bimodal” effect pattern, with both short-term and long-term interventions showing favorable outcomes, while the medium-term effect is relatively attenuated. Assessment of publication bias indicates that the results of most indicators are robust.

**Conclusion:**

Plaza dancing exerts significant positive effects on body composition and cardiopulmonary function in middle-aged and the aged healthy women, serving as a low-cost, highly accessible health promotion intervention. Future research should conduct large-sample, long-term follow-up studies to optimize intervention protocols and explore underlying mechanisms.

**Systematic review registration:**

www.crd.york.ac.uk/prospero, identifier CRD420251075375.

## 1 Introduction

With the acceleration of global aging processes, health issues among middle-aged and the aged populations have gradually become a focal point of social attention. According to World Health Organization estimates, by 2030, there will be over 1.2 billion menopausal women worldwide, with over 210 million menopausal women in China, accounting for approximately one-sixth of the total population. This enormous population faces unique health challenges, particularly body composition imbalance and cardiopulmonary dysfunction caused by physiological changes during menopause (1). From a physiological and pathological perspective, women entering perimenopause after age 45 experience significant decline in estrogen levels, leading to a series of cascading reactions, such as accelerated visceral fat accumulation (2); muscle mass reduction (3); and decreased vascular elasticity (4). In China, overweight (BMI ≥ 24 kg/m^2^) (5) and dyslipidemia (elevated cholesterol and triglycerides) have become major predictors of cardiovascular disease risk in this population, significantly increasing cardiovascular disease risk (6). Meanwhile, pulmonary function decline is associated with muscle mass reduction, leading to limitations in daily activity capacity and significant deterioration in quality of life (7). More seriously, these health problems often co-occur, forming multiple disease burdens that not only increase individual medical expenses but also impose heavy economic pressure on families and society (8). Therefore, finding effective health intervention strategies suitable for middle-aged and the aged women has important practical significance.

Current intervention approaches for body composition and cardiovascular function in middle-aged and the aged women mainly include pharmaceutical treatment and exercise training. Although pharmaceutical interventions have certain effects in some aspects, long-term use may bring side effects and dependency issues. For instance, hormone replacement therapy may increase the risk of cardiovascular complications and breast cancer (9, 10). Traditional exercise interventions such as running and equipment training, while capable of improving specific physical functions, often have the following limitations: exercise intensity is difficult to control precisely, easily causing excessive joint loading (11, 12); lack of enjoyment and sociability (13), resulting in low participation and adherence among middle-aged and the aged women; high requirements for venues and equipment, limiting large-scale promotion and application. These limitations highlight the importance of exploring novel, age-appropriate exercise intervention approaches.

Plaza dancing, as a widely practiced group physical activity in China, shows unique potential in addressing the limitations of traditional interventions. It features moderate exercise intensity and cyclic movement design that can continuously consume energy while avoiding excessive joint loading; musical rhythm regulates exercise tempo, facilitating maintenance of appropriate heart rate zones; collective synchronized movements enhance social belonging, significantly improving exercise enjoyment and persistence; simultaneously, it is not restricted by venues or equipment and can be performed in open spaces such as plazas and parks, offering advantages of low cost and easy promotion with high accessibility (14). These characteristics make plaza dancing an ideal candidate for health intervention among middle-aged and the aged women.

In recent years, existing research has shown that plaza dancing intervention has significant positive effects in improving physiological indicators of body composition and cardiopulmonary function (15). Regarding body composition, studies by Deng (16) and Li (17) both found that plaza dancing intervention could significantly reduce participants’ weight, body mass index, and body fat percentage. As research progressed, Zheng et al. (18) through a cross-sectional study of 342 middle-aged women, found that middle-aged women engaged in long-term plaza dancing were superior to long-term walkers in body morphology and exercise capacity, suggesting that plaza dancing may have better fitness effects, although more long-term intervention studies are still needed to verify its long-term effectiveness as a fitness method. Recent studies have paid more attention to the differential effects of exercise modalities. Ma et al. (19) found that the association between plaza dancing exercise duration and various body composition indicators in postmenopausal women was superior to that of brisk walking exercise, providing empirical evidence for exercise modality selection. Research on cardiopulmonary function improvement also shows positive results. Multiple studies have confirmed that plaza dancing can effectively improve participants’ resting heart rate, blood pressure, vital capacity, and other basic cardiopulmonary function indicators, and have beneficial effects on lipid metabolism (20, 21). More importantly, studies by Wu (22), Zhang (23), and Jia (24) confirmed from a disease prevention perspective that regular plaza dancing exercise has significant protective effects in reducing the incidence of cardiovascular diseases such as atherosclerosis and coronary heart disease. Furthermore, research on exercise prescription characteristics such as duration, frequency, and intensity of dance intervention (25, 26) has further enriched scientific understanding of plaza dancing exercise effects. Although existing reviews have reported evidence of the impact of dance interventions on fitness and physical health (27–29), for example, Fong Yan et al. (30) conducted a systematic review of 28 studies comparing the effects of structured dance interventions with other structured exercise programs on physical health outcomes, finding that dance was equally effective as traditional exercise in improving body composition, blood biomarkers, and musculoskeletal function. However, that study mainly involved traditional dance forms such as ballet, modern dance, and aerobic dance, with participants spanning a wide age range (covering all age groups). Meanwhile, there are obvious deficiencies in the following aspects: first, it did not specifically target plaza dancing as a specific exercise form; second, it did not focus on the high-risk special population of middle-aged and the aged women; third, it lacked systematic comprehensive assessment of body composition and cardiovascular function; fourth, there is still no high-quality systematic review or meta-analysis providing evidence-based medical evidence.

In summary, although plaza dancing shows good prospects in improving the health of middle-aged and the aged women, related research still has critical gaps: first, there is a lack of quantitative evidence-based evaluation of specific plaza dancing intervention effects; second, there is a lack of systematic analysis of the effect differences of different intervention parameters (duration, frequency, sample size, etc.); finally, there is a lack of comprehensive effect assessment from the dual dimensions of body composition and cardiovascular function. These research gaps limit the effective translation of plaza dancing from theoretical research to clinical application and hinder its scientific promotion in community health promotion. Therefore, this study aims to quantitatively assess for the first time the comprehensive impact of plaza dancing on body composition and cardiopulmonary function in healthy middle-aged and the aged women, systematically analyze the moderating effects of different intervention parameters, and provide scientific evidence for developing evidence-based plaza dancing health intervention programs. This study not only helps fill existing research gaps but also provides important evidence-based medical evidence for promoting this low-cost, highly accessible indigenous exercise intervention, with important theoretical value and practical significance.

## 2 Methods

This study was conducted following the Preferred Reporting Items for Systematic Reviews and Meta-Analyses (PRISMA) guidelines (31) and the Cochrane Collaboration Handbook (32). The protocol was registered in the International Prospective Register of Systematic Reviews (PROSPERO, CRD420251075375).

### 2.1 Literature search and study selection

To evaluate the effectiveness of plaza dancing interventions on health-related outcomes in middle-aged and the aged women, a systematic search was conducted across multiple databases, including PubMed, Web of Science, CNKI, Wanfang, and VIP. The search timeframe extended from database inception to July 3, 2025, with language restrictions limited to Chinese and English.

For plaza dancing interventions, search terms included: (“Square Dance”[Title/Abstract] OR “Square Fitness Dance”[Title/Abstract] OR “Plaza Dance”[Title/Abstract]).

For the target population of middle-aged and the aged women, search terms included: (“Middle Aged”[MeSH] OR “Female”[MeSH] OR “Aged”[MeSH] OR “Postmenopause”[MeSH] OR “Perimenopause”[MeSH] OR “Menopause”[MeSH] OR “Climacteric”[Title/Abstract]).

For health-related outcome indicators, search terms included: (“Body Composition”[MeSH] OR “Body Fat Percentage”[Title/Abstract] OR “Body Fat Rate”[Title/Abstract] OR“Body Weight”[MeSH] OR“Body Mass Index”[MeSH] OR “BMI”[Title/Abstract] OR “Heart Rate”[MeSH] OR “Resting Heart Rate”[Title/Abstract] OR “Quiet Heart Rate”[Title/Abstract] OR “Vital Capacity”[MeSH] OR “Pulmonary Function”[Title/Abstract] OR “Blood Pressure”[MeSH] OR “Systolic Blood Pressure”[Title/Abstract] OR “Cholesterol”[MeSH] OR “Total Cholesterol”[Title/Abstract] OR “Triglycerides”[MeSH] OR “Triacylglycerol”[Title/Abstract]).

The three groups of search terms were combined using the “AND” logical operator. Search terms were fine-tuned according to the characteristics of different databases to ensure comprehensive retrieval of relevant studies.

### 2.2 Study selection criteria

This study strictly followed the PICOS (Population, Intervention, Comparison, Outcome, Study design) strategy to develop inclusion and exclusion criteria for literature, ensuring the systematic and scientific nature of the study selection. The specific research selection criteria are shown in Table 1.

**Table 1:** Inclusion and exclusion criteria chart for literature.

PICOS components	Inclusion criteria	Exclusion criteria
Population	Healthy middle-aged and older women aged ≥45 years, without severe physical diseases, and not requiring prescribed medications that affect body composition and cardiovascular function	Young women (<45 years), males, middle-aged and older women with severe physical disabilities, and individuals with chronic diseases requiring long-term medication control
Intervention	Plaza Dance as the primary intervention modality, occurring in any suitable locations including plazas, parks, streets, and open spaces in front of buildings, with intervention content clearly described as Plaza Dance exercise	Plaza Dance combined with other types of interventions (such as dietary plans, other physical activities combined with Plaza Dance), or non-Plaza Dance types of dance interventions
Comparison	Studies with control groups where the control group does not participate in Plaza Dance, engages only in routine daily activities, or participates in non-Plaza Dance light activities	Studies without control group design, or studies where the control group engages in other types of exercise interventions but cannot distinguish the specific effects of Plaza Dance
Outcomes	Reports at least one body composition indicator (body weight, BMI, body fat percentage) and/or at least one cardiopulmonary function indicator (resting heart rate, systolic blood pressure, vital capacity, cholesterol, triglycerides), with quantitative data provided before and after intervention	Studies reporting only subjective health perceptions or quality of life scores, lacking objective body composition or cardiopulmonary function measurement indicators, or incomplete data that cannot be extracted for effective analysis
Study design	Randomized controlled trials (RCTs), cluster randomized trials, quasi-randomized controlled trials	Cross-sectional studies, case reports, studies published only in abstract, commentary or review format, or single-arm trials without control groups

### 2.3 Data extraction and preparation

A customized data extraction tool was designed in Excel (Microsoft Inc., Redmond, WA, USA) to extract data. To ensure the accuracy of literature deduplication, the study primarily used EndNote software for literature management and duplicate detection. Initially, duplicate literature was identified through EndNote’s automatic duplicate detection function, followed by manual verification by researchers to ensure removal of all duplicate records. Two researchers independently extracted data and cross-validated to ensure accuracy and completeness. Discrepancies were resolved by arbitration from a third researcher. Extracted data included study authors, population source, sample size, participant age, intervention details (dance type, protocol, frequency, duration), outcome measures and results (means and standard deviations). All data underwent double entry and cross-validation to minimize human error and enhance study reliability.

### 2.4 Study quality assessment

Two independent authors rigorously assessed the quality of all included studies. For randomized controlled trials (RCTs), the Cochrane Risk of Bias tool (ROB 2.0) was employed (33), evaluating overall bias risk based on scoring in domains including randomization process, deviations from intended interventions, missing outcome data, outcome measurement, and selective reporting. Studies were classified as “low risk” if all key domains were rated as low risk; “moderate risk” if at least one domain was rated as moderate risk with no key domains rated as high risk; and “high risk” if at least one key domain was rated as high risk or multiple non-key domains exhibited moderate or high risk. If consensus could not be reached, a third author intervened to make the final decision on bias risk rating. Subsequently, the research team graded the overall quality of included studies (low, moderate, high risk) based on bias assessment risk and explored the potential impact of bias on study result interpretation. This stratified evaluation framework ensured both objectivity and rigor in quality assessment while enhancing the reliability and persuasiveness of study results.

### 2.5 Statistical analysis

Meta-analysis was conducted on 17 of the 38 included studies to estimate the comprehensive effects of plaza dancing on body composition and cardiovascular function in middle-aged and the aged healthy women. Only studies providing necessary information for pre- and post-intervention measurements and having control groups were included in the meta-analysis. Meta-analyses were performed for two specific conditions: (1) body composition indicators (body weight, BMI, body fat percentage); and (2) cardiopulmonary function indicators (resting heart rate, systolic blood pressure, vital capacity, cholesterol, triglycerides). If studies reported multiple outcomes within each article, results for various indicators of body composition and cardiopulmonary function were all included.

Considering the different outcomes used across studies, statistical analyses were performed using Review Manager version 5.4. For included studies, standardized mean differences (SMD) or mean differences (MD) were calculated. When measurement tools differed between studies, SMD was used to standardize data for comparative analysis. When measurement tools were consistent, MD was used directly. For pooled analyses, random-effects models (I^2^ > 50%) or fixed-effects models (I^2^ < 50%) were employed based on the degree of heterogeneity assessed through I^2^ statistics. When heterogeneity exceeded 50%, subgroup analyses were conducted to explore sources of variation. Pre-specified sensitivity analyses were performed by omitting one study at a time to investigate the influence of individual studies on the overall pooled estimate.

## 3 Results

### 3.1 Literature search and characteristics of included studies

**Figure 1:**
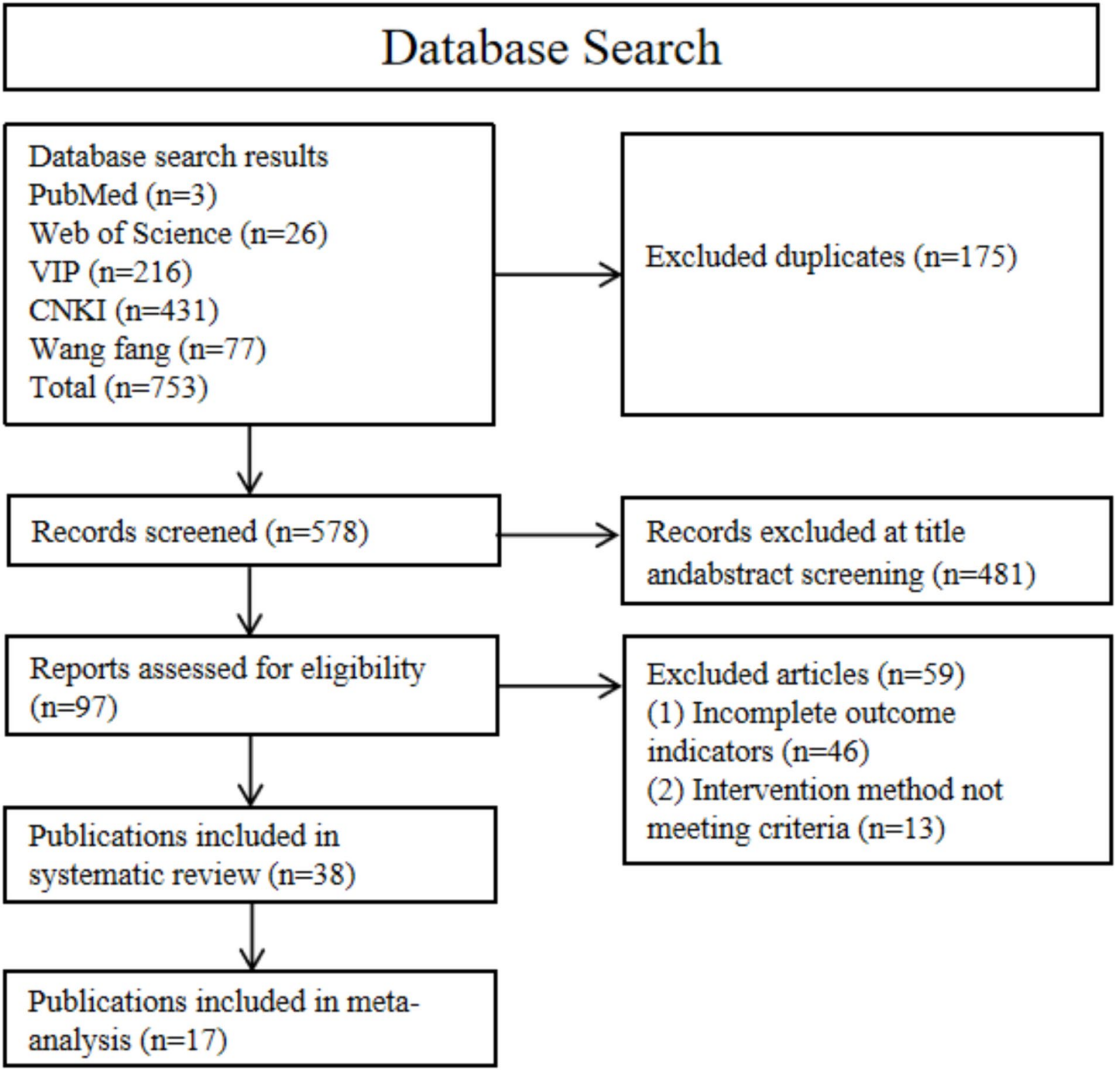
A flowchart of the literature search and study selection according to the PRISMA standard.

Our search yielded 753 articles (Figure 1). After removing 175 duplicates, 578 titles and abstracts were screened. Through screening of titles and abstracts, we excluded 481 records. The full texts of the remaining 97 articles were reviewed according to the selection criteria, and ultimately 38 studies were included in the systematic review (6, 8, 14, 16–25, 34–43), of which 17 studies provided sufficient quantitative data and were included in the meta-analysis (44–60). Figure 1 presents the flowchart of the database search process.

### 3.2 Characteristics of included studies

The 18 included studies were published between 2013 and 2024. Study sample sizes varied considerably, ranging from a minimum of 18 to a maximum of 129 participants. The study subjects mainly focused on healthy middle-aged and the aged women aged ≥45 years without serious physical diseases, with mean ages predominantly ranging from 45–75 years. All studies recruited female participants, reflecting the prevalence and applicability of plaza dancing among middle-aged and the aged women. The duration of plaza dancing interventions varied widely, ranging from 8 to 27 weeks, with intervention frequency ranging from 2 to 7 times per week, and training duration per session ranging from 40–60 min to 120 min. Various forms of plaza dancing were employed in the studies, including plaza dancing (different ethnic styles), daily plaza dancing activities, plaza fitness dancing, and plaza fitness exercises, reflecting the diversity of plaza dancing exercise forms. Regarding control group settings, 2 studies established control groups engaging in other types of physical activities (such as brisk walking and tai chi) (44, 45), 11 studies had control groups maintain unchanged daily lifestyle (46–54, 60), and 5 studies did not establish specific intervention measures (55–59). This diverse control group setup helps better evaluate the specific effects of plaza dancing interventions. In terms of measurement indicators, 1 study provided body composition-related results (BMI) (53), 4 studies provided cardiopulmonary function-related results (including heart rate, blood pressure, vital capacity, etc.) (44, 46, 47, 54), while 12 studies simultaneously assessed both body composition and cardiopulmonary function (44, 45, 48–52, 54–59). Measurements of body composition and cardiopulmonary function employed professional equipment including automated biochemical analyzers, body composition analyzers, pulmonary function instruments, and heart rate and blood pressure monitors. All included studies reported that plaza dancing had positive effects on body composition and cardiopulmonary function, thereby achieving improvement in overall health levels of middle-aged and the aged women. Studies included only in narrative synthesis also indicated that plaza dancing can effectively improve body composition and cardiopulmonary function levels in middle-aged and the aged women, providing evidence support for plaza dancing as an effective health promotion intervention. See Supplementary Table 1 for detailed descriptions of the specific content and protocols of plaza dancing interventions in the included studies.

### 3.3 Publication bias assessment

**Figure 2:**
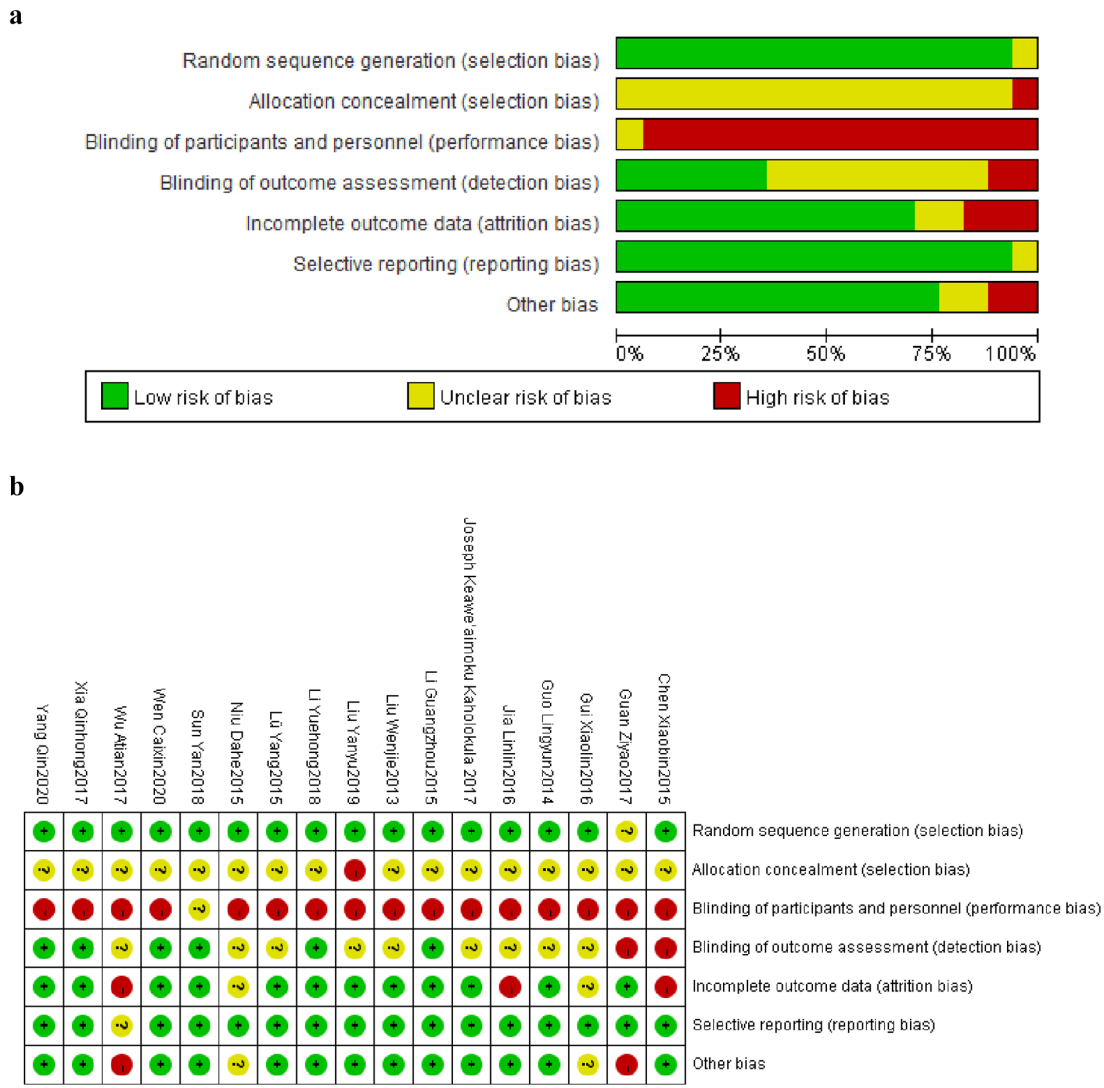
**(a)** Graphical map of RCT bias analysis. **(b)** Summary map of RCT bias analysis.

As shown in Figures 2a,b, based on Cochrane official recommended standards, this study used Review Manager 5.4 software combined with the latest bias risk assessment tool (ROB2.0) to systematically evaluate the 17 included randomized controlled trials. The assessment results showed: In terms of randomization process, 16 studies were rated as low risk, while 1 study was rated as unclear risk due to insufficient detail in describing the randomization process (49); Regarding deviation from intended interventions, 16 studies were rated as high risk due to inadequate blinding implementation, while 1 study was marked as unclear risk due to insufficient detail description (58); In terms of data completeness, 12 studies completely reported outcome data showing low risk, while 3 studies were rated as high risk due to data missing caused by low participant adherence (49, 53, 59); In the domains of outcome measurement and selective reporting bias, 6 studies were rated as low risk, while 9 studies were marked as unclear risk due to insufficient methodological description (43, 44, 48, 49, 52, 55–57, 59). Overall, the bias risk credibility of included RCT studies was relatively high. Although some unclear risk points existed, 1 study with insufficient detail in randomization process description showed lower risk in key domains such as confounding bias and selective reporting bias. These results indicate that the conclusions drawn from these studies are largely reliable.

### 3.4 Meta-analysis results

**Figure 3:**
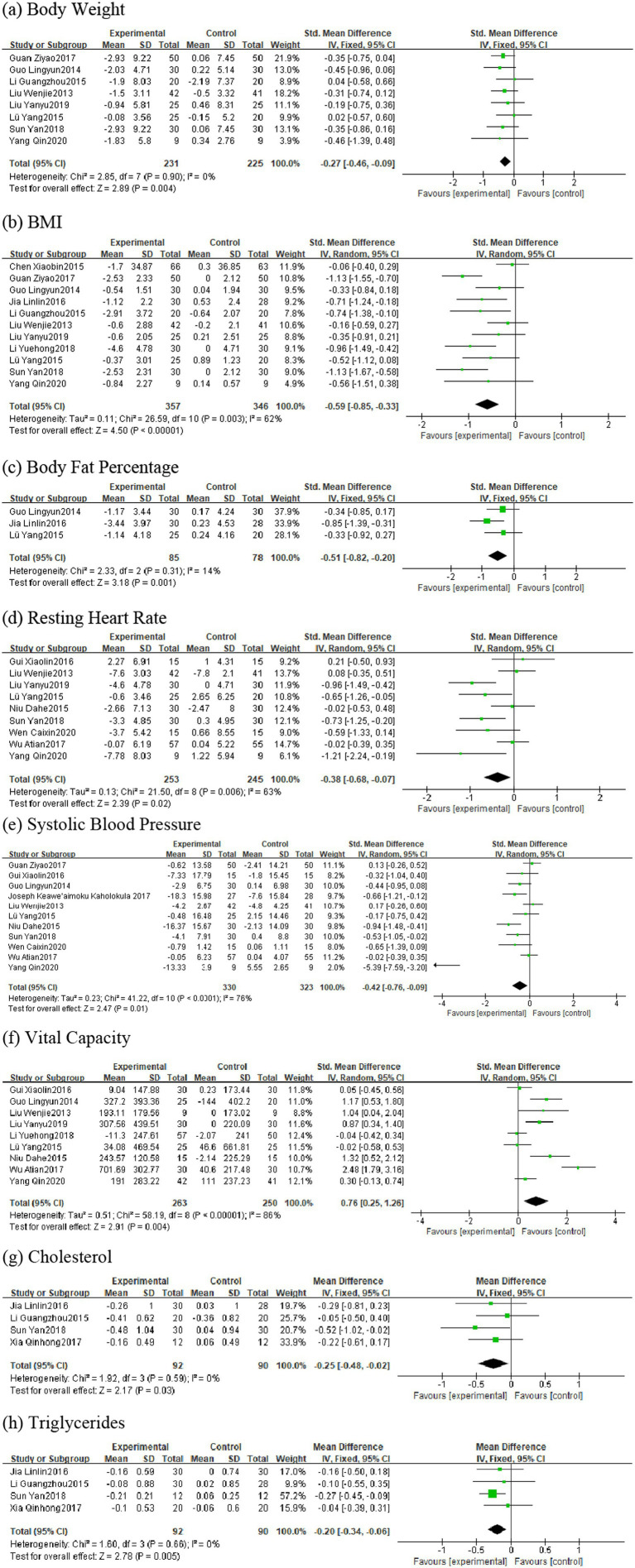
Forest plot for square dance vs. control group.

This meta-analysis ultimately included 17 studies involving 1,014 healthy middle-aged and the aged female participants aged 45 and above. Regarding the effects of plaza dancing intervention on body composition and cardiovascular function, we generated forest plots for eight core outcome indicators, including body composition indicators (body weight, BMI, body fat percentage) and cardiopulmonary function indicators (resting heart rate, systolic blood pressure, vital capacity, cholesterol, triglycerides). Based on heterogeneity assessment results, we also conducted subgroup analyses for four key indicators—BMI, resting heart rate, systolic blood pressure, and vital capacity—to explore effect modifying factors and explain sources of between-study differences. The characteristics of the meta-analysis data are detailed in Figure 3.

#### 3.4.1 Body composition indicators

##### 3.4.1.1 Body weight

Eight studies were included in the analysis (43, 44, 48, 50, 51, 56–58), with total sample sizes of 231 and 225 in the intervention and control groups, respectively. Heterogeneity testing showed I^2^ = 0%, *p* = 0.90, so a fixed-effects model was used for analysis. The pooled effect size results indicated that plaza dancing intervention significantly reduced body weight in healthy middle-aged and the aged women, with SMD of −0.27, 95%CI [−0.46, −0.09], *p* = 0.004. Low heterogeneity indicated high consistency between study results, demonstrating strong evidence robustness (Figure 3A).

##### 3.4.1.2 BMI

Eleven studies were included in the analysis (43, 44, 46, 48–51, 53, 56–58), with total sample sizes of 357 and 346 in the intervention and control groups, respectively. Heterogeneity testing showed I^2^ = 62%, *p* = 0.003, possibly related to differences in intervention frequency and measurement tools, so a random-effects model was used for analysis. The pooled effect size showed that plaza dancing intervention significantly reduced BMI in healthy middle-aged and the aged women, with SMD of −0.59, 95%CI [−0.85, −0.33], *p* < 0.00001. Subsequent subgroup analysis further explored sources of heterogeneity (Figure 3B).

##### 3.4.1.3 Body fat percentage

Three studies were included in the analysis (49, 56, 57), with total sample sizes of 85 and 78 in the intervention and control groups, respectively. Heterogeneity analysis showed I^2^ = 14%, *p* = 0.31, using a fixed-effects model. The standardized mean difference (SMD) was −0.51, 95%CI [−0.82, −0.20], *p* = 0.001, indicating that plaza dancing intervention significantly reduced body fat percentage in healthy middle-aged and the aged women, with statistically significant improvement effects (Figure 3C).

#### 3.4.2 Cardiopulmonary function indicators

##### 3.4.2.1 Resting heart rate

Nine studies participated in the analysis (44, 46, 51, 52, 54, 55, 57–59), with total sample sizes of 253 and 245 in the intervention and control groups, respectively. Heterogeneity testing showed I^2^ = 63%, *p* = 0.006, using a random-effects model. The pooled effect size showed SMD of −0.38, 95%CI [−0.68,−0.07], *p* = 0.02, indicating that plaza dancing has significant improvement effects on resting heart rate (Figure 3D).

##### 3.4.2.2 Systolic blood pressure

Eleven studies participated in the analysis (44, 50–52, 54–60), with total sample sizes of 330 and 323 in the intervention and control groups, respectively. Heterogeneity testing showed I^2^ = 76%, *p* < 0.0001, using a random-effects model. The pooled effect size showed SMD of −0.42, 95%CI [−0.76,−0.09], *p* = 0.01, indicating that plaza dancing has significant blood pressure-lowering effects with clear cardiovascular protective effects (Figure 3E).

##### 3.4.2.3 Vital capacity

Nine studies were included in the analysis (32, 34, 36, 39, 40, 43–45, 47), with total sample sizes of 263 and 255 in the intervention and control groups, respectively. Heterogeneity testing showed I^2^ = 86%, *p* < 0.0001, using a random-effects model. The pooled effect size showed SMD of 0.76, 95%CI [0.25, 1.26], *p* < 0.0001, suggesting that plaza dancing intervention significantly improved vital capacity with notable respiratory function improvement effects (Figure 3F).

##### 3.4.2.4 Cholesterol

Four studies were included in the analysis (43, 45, 49, 58), with total sample sizes of 92 and 90 in the intervention and control groups, respectively. Heterogeneity testing (I^2^ = 0%, *p* = 0.59) used a fixed-effects model. Results showed that plaza dancing intervention significantly reduced cholesterol levels, with MD = −0.25, 95%CI [−0.48,−0.02], *p* = 0.03. This magnitude of cholesterol reduction has clinical significance for cardiovascular disease risk prevention and control (Figure 3G).

##### 3.4.2.5 Triglycerides

**Figure 4:**
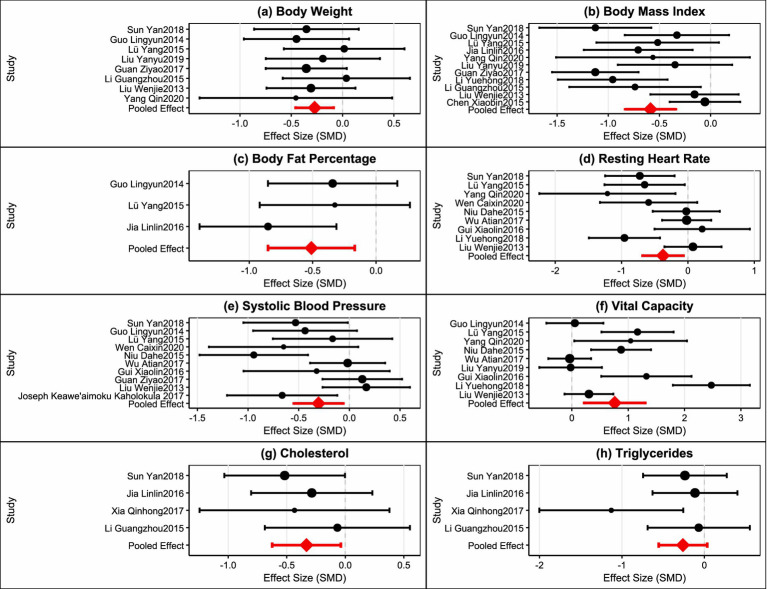
Sensitivity analysis plot.

Four studies were included in the analysis (43, 45, 49, 58), with total sample sizes of 92 and 90 in the intervention and control groups, respectively. Heterogeneity testing (I^2^ = 0%, *p* = 0.66) used a fixed-effects model. Results showed that plaza dancing intervention significantly reduced triglyceride levels, with MD = −0.20, 95%CI [−0.34,−0.06], *p* = 0.005, indicating that plaza dancing can effectively reduce triglyceride levels with clear positive effects on lipid metabolism (Figure 3H). Overall, body weight, body fat percentage, cholesterol, and triglyceride indicators showed low heterogeneity (I^2^ ≤ 50%) with good evidence homogeneity; while BMI, resting heart rate, systolic blood pressure, and vital capacity indicators exhibited moderate to high heterogeneity (I^2^ = 63–86%). To verify the robustness of the analysis results, we used leave-one-out analysis to assess the impact of each included study on the pooled effect size. Sensitivity analysis results showed (Figure 4) that most outcome indicators demonstrated good robustness. For body composition indicators (body weight, BMI, body fat percentage), after removing any single study, the pooled effect sizes and 95% confidence intervals showed no substantial changes, with consistent effect directions and stable statistical significance. Among cardiopulmonary function indicators, systolic blood pressure analysis identified one obviously outlying study (51) (SMD = −5.39). After removing this study, the pooled effect size adjusted from SMD = −0.42 to SMD = −0.30, heterogeneity decreased from I^2^ = 76% to I^2^ = 56%, but statistical significance remained stable (*p* = 0.01) with notably improved evidence quality. Other indicators such as resting heart rate and vital capacity all showed good stability, with small effect size changes after removing any single study. Blood lipid indicators had relatively low statistical power for sensitivity analysis due to limited number of included studies, but effect size change ranges were small with consistent directions. In summary, except for one outlier in the systolic blood pressure indicator, the main conclusions of this analysis have good robustness and do not depend on extreme results from individual studies, providing reliable evidence-based evidence for plaza dancing health intervention effects.

### 3.5 Publication bias

Publication bias is an important factor affecting the reliability of systematic review and meta-analysis results. This study used funnel plots, Egger regression tests, and trim-and-fill method to assess publication bias in the included studies.

**Figure 5:**
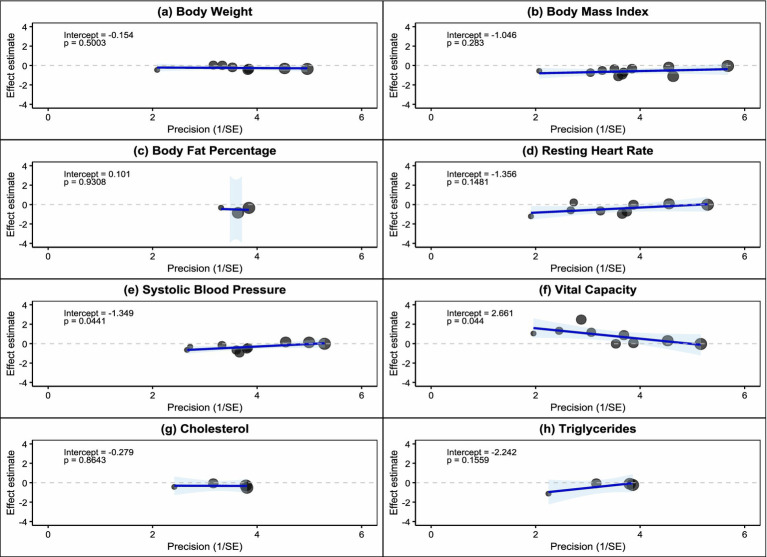
Contour-enhanced funnel plots of included studies.

Funnel plot analysis showed (Figure 5) that the distribution of study points for most outcome indicators was relatively symmetrical, conforming to the expected inverted funnel shape. Study points for body weight, BMI, body fat percentage, cholesterol, and triglycerides were basically symmetrically distributed around the effect estimate, with no obvious asymmetry observed. However, systolic blood pressure and vital capacity indicators showed slight asymmetric distribution patterns, with study points skewed toward the side with larger effect sizes, suggesting possible publication bias.

**Figure 6:**
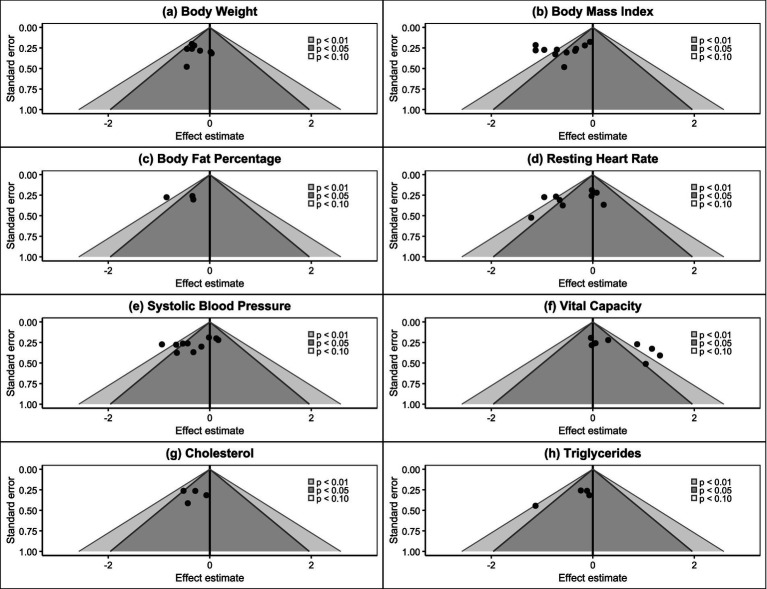
Egger regression test included studies.

Egger regression tests further quantified the degree of publication bias (Figure 6). Results showed that body weight (intercept = −0.154, *p* = 0.5003), BMI (intercept = −1.046, *p* = 0.283), body fat percentage (intercept = 0.101, *p* = 0.9308), resting heart rate (intercept = −1.356, *p* = 0.1481), cholesterol (intercept = −0.279, *p* = 0.8643), and triglycerides (intercept = −2.242, *p* = 0.1559) all had Egger test *p*-values greater than 0.05, indicating no significant publication bias for these indicators. However, systolic blood pressure (intercept = −1.349, *p* = 0.0041) and vital capacity (intercept = −2.661, *p* = 0.041) showed statistically significant Egger tests, suggesting the presence of publication bias. The negative intercept values indicate that small-sample studies tend to report larger negative effects, possibly reflecting the phenomenon of lower publication probability for studies with negative results.

**Figure 7:**
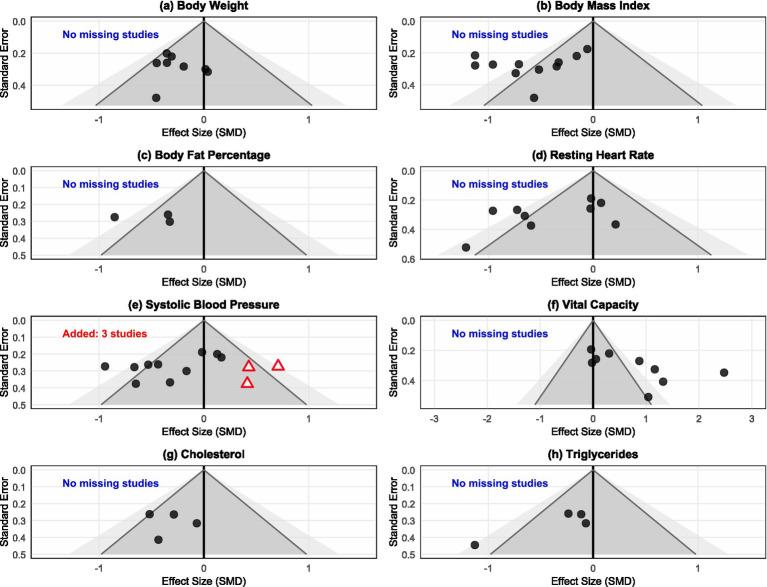
Results of trim-and-fill analysis for publication bias assessment.

Trim-and-fill analysis results showed (Figure 7) that the systolic blood pressure indicator required the addition of 3 “missing” studies (marked with red triangles) after trim-and-fill adjustment, while all other indicators showed “No missing studies,” indicating no need to supplement missing studies. This result was consistent with the Egger test results, further confirming the possibility of publication bias in the systolic blood pressure indicator. Comprehensive assessment indicated that 6 outcome indicators (75%) in this meta-analysis showed no significant publication bias, with reliable evidence quality. Although systolic blood pressure and vital capacity indicators showed some publication bias, considering: (1) the relatively limited number of included studies; (2) overall small effect sizes; (3) sensitivity analysis showing robust results, the impact of this bias on the overall conclusions of the meta-analysis remains within acceptable limits. Therefore, the main conclusions of this study have good credibility, but caution should be exercised when interpreting results related to systolic blood pressure and vital capacity.

### 3.6 Subgroup analysis

**Figure 8:**
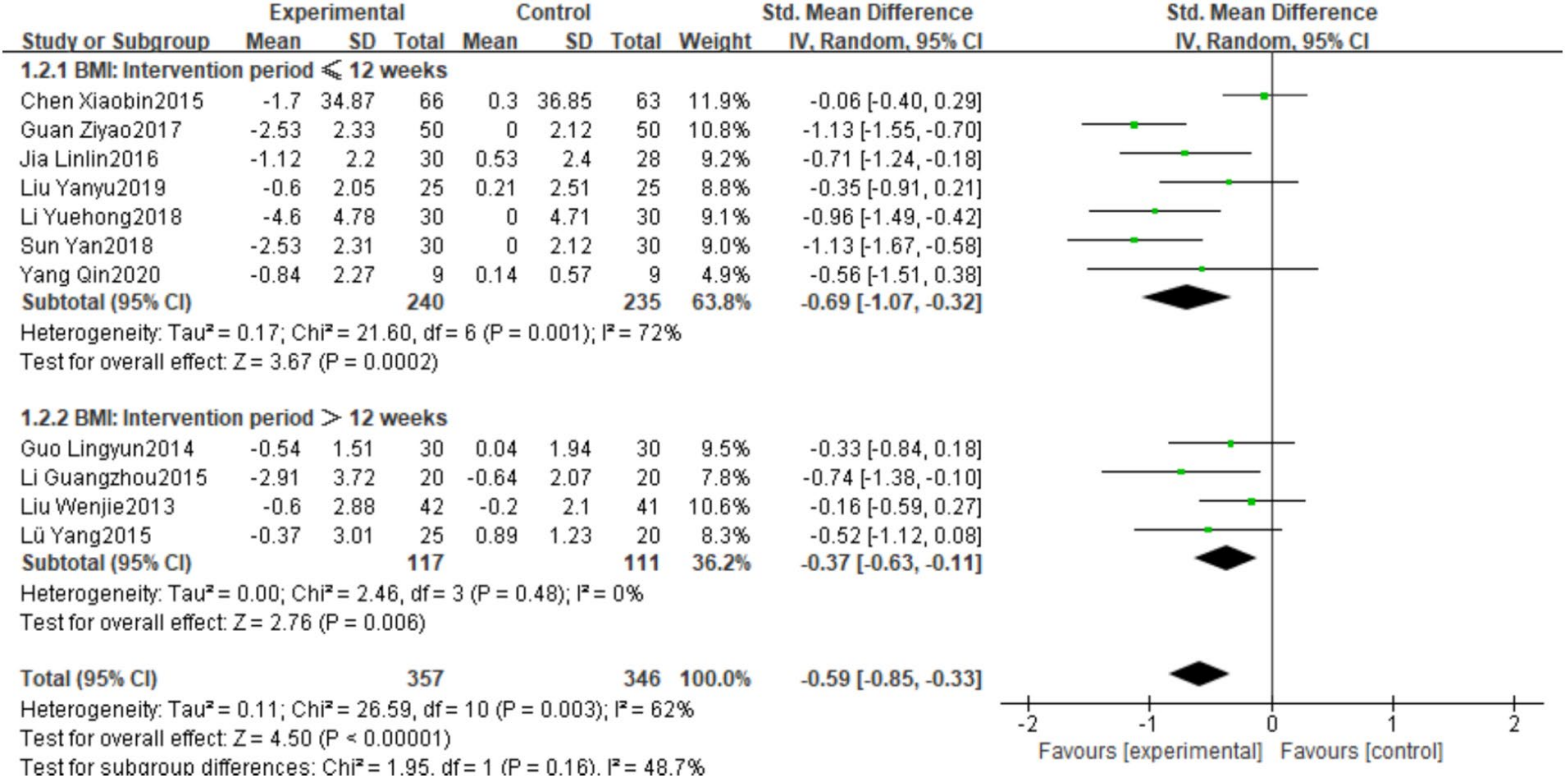
Subgroup analysis of plaza dancing intervention effects on BMI.

Based on the aforementioned meta-analysis results, we found that four key indicators—BMI, resting heart rate, systolic blood pressure, and vital capacity—exhibited moderate to high heterogeneity (I^2^ = 63–86%). To deeply explore sources of heterogeneity and identify key factors affecting plaza dancing intervention effects, we conducted predetermined subgroup analysis for the BMI indicator, as shown in Figure 8.

BMI subgroup analysis using 12 weeks as the cutoff point found (Figure 8) that the intervention duration ≤12 weeks group showed more significant improvement effects (SMD = −0.69, 95%CI [−1.07, −0.32], *p* < 0.05), but with higher heterogeneity (I^2^ = 72%); the intervention duration >12 weeks group showed relatively smaller improvement effects (SMD = −0.37, 95%CI [−0.63, −0.11], *p* < 0.05), with significantly reduced heterogeneity (I^2^ = 0%). These results suggest that short-term plaza dancing intervention may be more effective in improving BMI change magnitude.

### 3.7 Meta-regression analysis and dose–response relationship

To further explore the potential dose–response relationship between plaza dancing intervention effects and intervention duration, this study employed meta-regression analysis methods, using intervention duration (weeks) as a continuous variable and standardized mean difference (SMD) or mean difference (MD) as dependent variables, constructing linear and quadratic regression models for quantitative assessment.

#### 3.7.1 Body composition indicators

Meta-regression analysis of body weight indicators included 8 studies (intervention time 8–27 weeks) (Supplementary Figure 1a), with linear regression showing no significant relationship between intervention duration and body weight improvement effects (*β* = 0.003, 95%CI [−0.020, 0.026], *p* = 0.817, R^2^ = 0.000). BMI indicators included 11 studies (8–27 weeks) (Supplementary Figure 1b), showing a significant negative correlation (*β* = 0.043, 95%CI [0.025, 0.061], *p* < 0.001), with extremely high heterogeneity explanation (R^2^ = 0.998). Short-term interventions (8–12 weeks) had larger effect sizes (SMD approximately −1.0 to −1.2), while long-term interventions (24–27 weeks) had significantly smaller effect sizes (SMD approximately −0.1 to −0.2). Body fat percentage indicators included 3 studies (12–24 weeks) (Supplementary Figure 1c), showing a negative correlation trend but not reaching significance (*β* = 0.046, 95%CI [−0.014, 0.106], *p* = 0.122, R^2^ = 0.000).

#### 3.7.2 Cardiopulmonary function indicators

Meta-regression analysis of resting heart rate included 9 studies (8–27 weeks) (Supplementary Figure 1d), showing a significant negative correlation (*β* = 0.041, 95%CI [0.010, 0.072], *p* = 0.011, R^2^ = 0.040). Short-term interventions showed larger negative effect sizes (SMD approximately −0.8 to −1.3), while long-term intervention effect sizes approached zero. Systolic blood pressure included 11 studies (8–27 weeks) (Supplementary Figure 1e), showing a negative correlation trend but not reaching significance (β = 0.021, 95%CI [−0.020, 0.062], *p* = 0.302, R^2^ = 0.088). Vital capacity included 9 studies (8–27 weeks) (Supplementary Figure 1f), with negative correlation trend approaching significance (β = −0.055, 95%CI [−0.119, 0.009], *p* = 0.092), and relatively high heterogeneity explanation (R^2^ = 0.475). Cholesterol indicators included 4 studies (8–24 weeks) (Supplementary Figure 1g), showing no obvious time dependency (β = 0.013, 95%CI [−0.059, 0.085], *p* = 0.688, R^2^ = 0.000). Triglycerides included 4 studies (8–24 weeks) (Supplementary Figure 1h), presenting a unique pattern: short-term intervention effects were moderate (SMD ≈ −0.3), mid-term effects were weakened (SMD ≈ −0.1), and long-term intervention effects were significantly enhanced (SMD ≈ −1.2), with quadratic regression model approaching significance (*p* = 0.093), showing a U-shaped reversal trend. Meta-regression analysis showed that BMI and resting heart rate had significant negative time effects, vital capacity approached significance, while body weight, body fat percentage, systolic blood pressure, and cholesterol showed no significant time dependency. Triglycerides presented a special “bimodal” effect pattern. Regarding heterogeneity explanation capability, the BMI regression model performed best (R^2^ = 0.998), the vital capacity model explained moderate heterogeneity (R^2^ = 0.475), while other indicators had limited explanatory capability.

## 4 Discussion

The meta-analysis results demonstrate that plaza dancing produced statistically significant positive effects in improving body weight, BMI, body fat percentage, resting heart rate, systolic blood pressure, vital capacity, and blood lipid indicators, providing evidence-based medical evidence for plaza dancing as a community health promotion intervention. These findings are consistent with previous research results on the health effects of exercise interventions, further supporting the application value of plaza dancing in health management for middle-aged and the aged women. Based on widely accepted exercise science principles, plaza dancing, as a low-intensity periodic aerobic exercise, may produce the observed health effects through multiple physiological pathways. It should be noted that since none of the 17 RCTs included in this study measured relevant physiological markers, the following mechanistic discussion is based on speculation from existing exercise physiology knowledge and lacks direct biological validation. Regarding body composition improvement, the sustained energy consumption pattern may theoretically affect fat oxidation processes, hypothetically improving fat metabolism efficiency (61). However, this study did not measure fat oxidase activity, metabolic rate, or related metabolic markers, making it impossible to verify this speculation. Second, the rhythmic pattern of plaza dancing may help maintain appropriate heart rate zones (60–80% maximum heart rate) (17, 48), theoretically ensuring energy consumption while potentially avoiding excessive stress responses (62). However, this study lacks objective exercise intensity monitoring data and stress hormone level measurements to confirm this hypothesis. Regarding cardiovascular function, based on general findings from previous sports medicine research, regular aerobic exercise may theoretically regulate the antioxidant system, hypothetically reducing oxidative stress responses and potentially improving vascular function (63); it may also affect autonomic nervous system balance, theoretically improving heart rate variability and parasympathetic regulation capacity (64). However, this study completely lacks vascular endothelial function markers (such as endothelin-1, nitric oxide), oxidative stress indicators, antioxidant enzyme activity, or heart rate variability analysis to verify these hypothetical mechanisms. Regarding blood lipid metabolism, Ben et al. (65) research on other dance forms suggests that such exercise may affect lipoprotein metabolism, hypothetically regulating lipoprotein lipase activity and potentially affecting hepatic lipid synthesis (66), theoretically reducing arterial wall lipid deposition (67). However, this study did not include lipoprotein lipase activity determination, liver function assessment, or detailed lipoprotein subtype analysis to confirm this speculation. Finally, regular coordinated exercise may theoretically help enhance respiratory muscle function, hypothetically improving pulmonary ventilation efficiency (68), however, this study lacks respiratory muscle strength testing, detailed pulmonary function assessment, or respiratory physiological indicator analysis to verify this hypothesis.

Meta-regression analysis further revealed the time-dependent characteristics of plaza dancing intervention effects. The BMI indicator showed a significant negative dose–response relationship (*p* < 0.001, R^2^ = 0.998), indicating that short-term intensive interventions (8–12 weeks) produced greater improvements than long-term interventions. This finding reveals an obvious “optimal intervention window” phenomenon for BMI improvement effects, where short-term interventions can produce maximum BMI reduction effects (SMD approximately −1.0 to −1.2), while the marginal benefits of extending intervention time diminish (SMD approximately −0.1 to −0.2). This pattern may reflect the physiological adaptation process of the body to plaza dancing intervention, including metabolic rate adjustment and re-establishment of energy balance. Resting heart rate also demonstrated significant negative time effects (*p* = 0.011), with short-term interventions (8–10 weeks) showing significant negative effect sizes (SMD approximately −0.8 to −1.3), while long-term intervention (24–27 weeks) effect sizes approached zero. This conforms to the adaptation patterns of the cardiovascular system to training stimuli, suggesting that 8–12 weeks may be the “golden window period” for cardiovascular function improvement. Notably, the triglyceride indicator presented a unique “biphasic” effect pattern, distinctly different from the monotonic trends of other indicators. Short-term intervention (8 weeks) showed moderate effects (SMD ≈ −0.3), mid-term intervention (12–16 weeks) showed weakened effects (SMD ≈ −0.1), while long-term intervention (24 weeks) showed significantly enhanced effects (SMD ≈ −1.2), with the quadratic regression fitting line showing an obvious U-shaped reversal trend. This unique pattern may reflect the complex physiological mechanisms of triglyceride metabolism. Early intervention mainly improves triglyceride levels through direct dietary and exercise effects, the mid-term may face metabolic adaptation and effect attenuation, while long-term intervention may activate deep-level lipid metabolism remodeling mechanisms, including liver function improvement, insulin sensitivity enhancement, and VLDL metabolism optimization. From the perspective of heterogeneity explanation capability, different indicators showed obvious differences. The BMI regression model performed most excellently (R^2^ = 0.998), essentially completely explaining between-study differences, indicating that intervention duration is the main source of BMI heterogeneity. The vital capacity regression model could explain nearly half of the between-study heterogeneity (R^2^ = 0.475), while between-study differences for other indicators may stem more from other factors such as intervention intensity, population characteristics, and measurement methods.

Subgroup analysis using 12 weeks as the cutoff point showed that the BMI improvement effect size for the ≤12 weeks intervention group (SMD = −0.69) was greater than the >12 weeks group (SMD = −0.37). This pattern may reflect the combined effects of physiological adaptation characteristics and practical implementation challenges. From the physiological adaptation perspective, the early stage of exercise training typically shows rapid neural adaptation and metabolic adjustment (69), followed by the body entering an adaptation plateau phase, where the improvement magnitude per unit time tends to flatten (70), which may explain the smaller effect size of the >12 weeks group (SMD = −0.37). Meanwhile, the extremely low heterogeneity in the >12 weeks group (I^2^ = 0%) indicates that long-term intervention studies have higher methodological consistency. The ≤12 weeks intervention group showed higher heterogeneity (I^2^ = 72%), while the >12 weeks group showed significantly reduced heterogeneity (I^2^ = 0%). This difference may be related to research implementation quality and participant adherence. Although dropout rate data in the included literature was limited, two studies in the ≤12 weeks group showed enormous dropout rate differences (3% vs. 35.5%). The effects of high dropout rate studies may be diluted, while low dropout rate studies may better reflect effects under ideal implementation conditions. From a behavioral perspective, long-term interventions may face challenges of declining participant adherence and motivation attenuation, which also partially explains the smaller effect sizes of long-term interventions. Sample size subgroup analysis showed that small sample studies (both groups ≤30) demonstrated larger effect sizes in resting heart rate, systolic blood pressure, and vital capacity (SMD of −0.54, −0.89, 0.97, respectively). However, this phenomenon mainly reflects methodological limitations of small sample studies, including publication bias, selection bias, effect overestimation due to insufficient statistical power, and incidental extreme results, rather than true biological effect advantages. Additionally, small sample studies typically lack sufficient statistical testing power to detect true but small effects. Therefore, sample size differences should not be interpreted as guidance for intervention implementation strategies. This finding mainly suggests that the meta-analysis results may be influenced by small sample effects and publication bias. Sensitivity analysis confirmed the robustness of this study’s results. One study (51) in the systolic blood pressure indicator showed an abnormally large effect size (−5.39). After removing this outlier, heterogeneity significantly decreased and the effect size remained statistically significant, indicating that plaza dancing’s improvement effects on blood pressure do not depend on individual extreme research results. Other indicators all maintained stable effect sizes and confidence intervals, further supporting the consistency of plaza dancing intervention effects and the reliability of research conclusions. It is worth noting that the health promotion effects of plaza dancing may have certain cultural specificity. As a collective exercise form rooted in Chinese culture, plaza dancing’s high participation rate and good adherence largely benefit from China’s collectivist cultural atmosphere and “plaza culture” tradition. Research shows that collective belonging and social support are key factors affecting exercise persistence rates in middle-aged and the aged women, and this social psychological effect may be weakened in individualistic cultural contexts (71). Additionally, plaza dancing music rhythms often use traditional Chinese folk music or popular songs, and cultural familiarity may enhance participants’ exercise enjoyment, thereby improving intervention effects. Therefore, when considering plaza dancing as an international health intervention strategy, it is necessary to fully assess the cultural acceptance of target populations and consider necessary cultural adaptability adjustments.

Based on meta-regression analysis results, this study provides certain reference bases for developing individualized plaza dancing intervention programs. Analysis results for body composition and cardiovascular indicators suggest that 8–12 week short-term intensive interventions may have better effects, which may help achieve relatively ideal cost-effectiveness ratios. For middle-aged and the aged women whose main goals are BMI and cardiovascular health improvement, an 8–12 week intensive intervention model could be considered. It is worth noting that after exceeding this time window, intervention strategies may need adjustment, with a recommended shift in focus to maintenance training. However, for patients with dyslipidemia, particularly elevated triglycerides, based on current limited evidence, longer-term sustained intervention (24 weeks and above) may be needed to achieve better effects. These findings partially explain sources of between-study heterogeneity and help enhance the reference value of meta-analysis results. In clinical practice, it is recommended to carefully consider developing differentiated intervention durations based on individual major health problems and target indicators to achieve better health promotion effects. However, this study also has certain limitations that require cautious interpretation of results. First, the 17 RCT studies included in the meta-analysis generally had small sample sizes (individual study sample sizes 18–129 people), which may affect the statistical power and generalizability of results. Second, some key indicators (vital capacity, systolic blood pressure) showed high heterogeneity, related to differences in measurement tool precision, intervention standardization degree, and population baseline characteristics across studies. Third, 16 studies could not achieve participant blinding due to the special nature of exercise intervention, presenting high bias risk in the “deviation from intended intervention” domain, which may lead to overestimation of intervention effects due to placebo effects, seriously affecting the strength of causal inference. Fourth, Egger tests showed significant publication bias for systolic blood pressure (*p* = 0.0013) and vital capacity (*p* = 0.041), suggesting possible missing studies with ineffective or negative results, leading to overestimation of effects for these indicators. Fifth, most studies did not report participant dropout rates and adherence data in detail, limiting assessment of true intervention effects. At the same time, studies lacked detailed physiological indicators such as fat oxidase activity, metabolic rate, or related metabolic markers, and follow-up times were generally short (mostly <6 months), making it difficult to assess long-term health effects and safety of plaza dancing. Additionally, the phenomenon observed in subgroup analysis that small sample studies showed larger effect sizes may be influenced by publication bias and small sample effects, suggesting that some of our conclusions may have overestimation risks, particularly recommendations regarding intervention implementation methods. More importantly, this study has significant cultural transferability limitations. The 17 RCT studies included mainly came from mainland China, and this geographical concentration limits the global applicability of research results. Plaza dancing, as a collective exercise form with Chinese cultural characteristics, has social attributes, music rhythms, and movement designs that are deeply integrated with Chinese cultural elements. Middle-aged and the aged women from different cultural backgrounds may have significant differences in acceptance, participation motivation, and persistence rates for collective dancing. Furthermore, systematic differences in lifestyle, dietary structure, genetic background, and common disease spectrum among populations in different regions, as well as differences in social support networks for plaza dancing in different cultural environments, may all affect the magnitude and sustainability of intervention effects. Therefore, when generalizing research results to other cultural backgrounds and geographical regions, cultural adaptability factors must be fully considered, and research conclusions cannot be directly extrapolated. Based on current findings and limitations, future research should deepen and improve in the following directions. First, conduct multi-center large sample randomized controlled trials with extended follow-up to 12–24 months, detailing participants’ adherence, dropout rates, and adverse events to accurately assess long-term effects and safety of plaza dancing intervention. Second, include detailed body composition indicators (muscle mass, bone density), vascular function markers (endothelial function, arterial stiffness), and metabolomics indicators to comprehensively evaluate the multi-dimensional health effects of plaza dancing. Simultaneously, combine molecular biology techniques to detect key metabolic enzyme activity, inflammatory factors, and vascular endothelial function markers to elucidate health promotion mechanisms at the molecular level. Finally, cross-cultural adaptability research is an important direction for future studies. It is recommended to conduct multi-center international collaborative research to verify the applicability and effect consistency of plaza dancing among middle-aged and the aged women from different cultural backgrounds. At the same time, based on different cultures’ music preferences and traditional dance elements, develop culturally adaptive dance variants and analyze their physiological effect differences among different ethnic populations. Through qualitative research, deeply understand participation barriers and promoting factors for middle-aged and the aged women from different cultural backgrounds, providing bases for developing cross-cultural promotion strategies, thereby providing evidence-based support for health promotion strategies in the global aging society.

## 5 Conclusion

This systematic review and meta-analysis evidence demonstrates that plaza dancing has significant improvement effects on lipid metabolism regulation, vascular function optimization, and respiratory function enhancement in healthy middle-aged and the aged women. Although subgroup analysis revealed that short-term studies show positive effects, long-term high-quality studies are needed to confirm the lasting health benefits of plaza dancing. Sensitivity analysis confirmed the robustness of the research results. Except for individual outliers, the effect sizes of all indicators remained stable and statistically significant, further supporting the effectiveness and reliability of plaza dancing as an exercise intervention for healthy middle-aged and the aged women. However, considering the relatively high risk of bias and publication bias existing in some outcomes, the conclusions of this study should be interpreted with caution. Although evidence suggests that plaza dancing has positive effects on healthy middle-aged and the aged women, more high-quality, rigorously designed studies are needed to confirm these effects. Furthermore, given that the evidence in this study mainly derives from the Chinese cultural sphere, the applicability of research results in other cultural backgrounds requires specific cross-cultural adaptability studies for verification. Despite the limitations in cultural transferability, this study provides a solid evidence-based medical foundation for the clinical application and policy promotion of plaza dancing.

## Data Availability

The original contributions presented in the study are included in the article/Supplementary material, further inquiries can be directed to the corresponding author.
