# Supplementary material for: Effects of plaza dancing on body composition and cardiopulmonary function in middle-aged and the aged healthy women: a systematic review and meta-analysis
Source: Front Public Health. 2025 Sep 8;13:1667818. doi: 10.3389/fpubh.2025.1667818 (PMC12450655; doi:10.3389/fpubh.2025.1667818)
Supplement: Supplementary file 1 [file Supplementary_file_1.docx]

Supplementary Table 1. A list of basic characteristics of the studies included in the meta-analysis.

| Study ID and Yea | Sample (EG/CG) | Average Age | Type of Intervention | Characteristics of Square Dance Content | Exercise Intervention Intensity | Exercise Intensity Monitoring | Intervention Cycle | Weekly Intervention Frequency | Duration of Intervention | Outcomes |
| --- | --- | --- | --- | --- | --- | --- | --- | --- | --- | --- |
| Sun Yan,2018 | 30/30 | 62.20±8.01/63.93±6.81 | Square dancing/No organized exercise | Progressive square dance training: basic step practice, dance movements (with music tempo from slow to fast), and relaxation movements | Progressive moderate intensity: Initial intensity gradually increases from 50% HRmax to the target intensity of 60%-70% HRmax | Real - time heart rate monitoring: Real - time monitoring with a Polar heart rate watch, target heart rate of 110 - 130 beats/min | 8weeks | 3 times a week | 60min | BW, BMI, SBP, RHR, VC, TG, TC |
| Guo Lingyun,2014 | 30/30 | 61.73±5.12/61.73+5.12 | Square dancing/No organized exercise | Internet - popular square dance training: high - click - rate square dances commonly used by middle - aged and elderly people, selected from Baidu and on - site observation | Moderate intensity: Exercise heart rate controlled within 50%-60% to 60%-80% of maximum heart rate | Intermittent self - monitoring: Measure the 10 - second heart rate during and after exercise respectively, allowing subjects to control exercise intensity by checking their pulse | 24weeks | 3 times a week | 50min | BW, BMI, BF%, RHR, SBP,VC |
| Lü Yang,2014 | 25/20 | 53.68±6.17/53.40±4.22 | Square dancing/No organized exercise | Internet - popular square dance training: high - click - rate square dances commonly used by middle - aged and elderly people, selected from Baidu and on - site observation | Moderate intensity: Exercise intensity maintained at approximately 60% of maximum heart rate | Intermittent monitoring: Control exercise load intensity through regular pulse tests | 24weeks | 4 times a week | 120min | BW, BMI, BF%, RHR, SBP,VC |
| Jia Linlin,2016 | 30/28 | 58.9±6.7/59.0±6.5 | Square dancing/Daily activities | Standardized square dance training: 12 sets of square fitness exercises launched by the General Administration of Sport of China | Moderate intensity: 66%-74% of maximum heart rate (calculated by the formula "220 - age") | Real - time heart rate monitoring: Continuous monitoring with a Polar heart rate watch, target heart rate of 100 - 120 beats/min | 12weeks | 5 times a week | ＞30min | BW, BMI, BF%, TC, TG |
| Yang Qin,2020 | 9/9 | 57.44±5.003/57.44±5.003 | Square dancing/Daily activities | Standardized square dance training: 12 sets of square fitness exercises launched by the General Administration of Sport of China | Moderate intensity: 50%-60% → 60%-80% HRmax, combined with the RPE scale (Level 11-14), corresponding to a heart rate of 110-140 beats per minute | Real - time heart rate monitoring: Real - time heart rate monitoring with a sports bracelet, target heart rate of 110 - 140 beats/min | 8weeks | 4 times a week | 40-60min | BW, BMI, RHR, SBP,VC |
| Wen Caixin,2020 | 15/15 | 63.87±1.55/62.83±1.47 | Square dancing/Daily activities | Integrated and innovative square dance training: collective square dances integrating elements of folk dances and aerobics are selected for the group | Moderate intensity: RPE scale Level 11-14, corresponding to a heart rate of 110-140 beats per minute | Real - time heart rate monitoring: Real - time heart rate monitoring with a sports bracelet, target heart rate of 110 - 140 beats/min | 12weeks | ＞3 times a week | 50-60min | RHR, SBP |
| Niu Dahe,2015 | 30/30 | 60.45±4.75/60.45±4.75 | Square dancing/No organized exercise | Personalized square dance training: square dances are self - organized collective square dances | Moderate to moderate-high intensity: Target heart rate is 60%-80% of maximum heart rate | Real - time heart rate monitoring: Real - time heart rate monitoring with a sports bracelet, target heart rate of 110 - 140 beats/min | 20weeks | ＞4 times a week | ＞60min | BW, BMI, RHR, SBP,VC |
| Wu Atian,2017 | 57/55 | 61.73±5.12/61.73+5.12 | Square dancing/No organized exercise | Personalized square dance training: songs are selected according to the participants' previous participation methods and preferences | Moderate-low intensity aerobic exercise | — | 24weeks | 5 times a week | 90min | BMI, RHR, SBP,VC |
| Xia Qinhong,2017 | 12/12 | 60-70 | Square dancing/Daily activities | Standardized square dance training: 12 sets of square fitness exercises launched by the General Administration of Sport of China | Relative exercise intensity: [(Immediate post-exercise heart rate － Resting heart rate) / (Maximum heart rate － Resting heart rate)] × 100% | Intermittent self - monitoring: Measure the 10 - second pulse during and after exercise, guiding subjects to adjust intensity independently | 24weeks | 5-7 times a week | 70min | TG , TC |
| Liu Yanyu,2019 | 25/25 | 45-59 | Square dancing/Daily activities | Music feature - oriented training: standardized movement combinations accompanied by soothing music | Moderate intensity: 55%-75% of maximum heart rate | Combined monitoring: Regular pulse tests + Subjective Perceived Exertion Scale | 12weeks | ＞4 times a week | ＞60min | BW, BMI,VC |
| Gui Xiaolin,2016 | 15/15 | 48±6.24/50.07±6.97 | Square dancing/Daily activities | Music feature - oriented training: standardized movement combinations accompanied by soothing music | Moderate intensity: Target heart rate of 110-140 beats per minute | Intermittent self - monitoring: Measure the 10 - second pulse during and after exercise, guiding subjects to adjust intensity independently | 12weeks | 3-5 times a week | 60min | BW, RHR,SBP,VC |
| Chen Xiaobin,2015 | 66/63 | 60-75 | Square dancing/Daily activities | Music feature - oriented training: standardized movement combinations accompanied by soothing music | Moderate-low intensity aerobic exercise | — | 24weeks | ＞4 times a week | 90min | BMI |
| Guan Ziyao,2017 | 50/50 | 64.3±5.6/65.4±6.9 | Square dancing/Daily activities | Traditional culture square dance training: group practice of traditional dances and specific controlled rhythm movements | — | — | 12weeks | ＞3 times a week | ＞60min | SBP, BMI |
| Li Yuehong,2018 | 30/30 | 62.5±2.5/63.30±3.1 | Square dancing/Daily activities | Standardized square dance training: 12 sets of square fitness exercises launched by the General Administration of Sport of China | Progressive moderate intensity: Low intensity in the initial stage increased to 60%-70% HRmax after 1-2 weeks and maintained | Equipment monitoring: Portable cardiac function detector AF930, monitoring 60% - 70% HRmax | 10weeks | 3-5 times a week | 60-90min | VC |
| Li Guangzhou,2015 | 20/20 | 59.12±2.64/61.69±6.87 | Square dancing/Fitness walking | Personalized square dance training: square dances are self - organized collective square dances | Moderate to moderate-high intensity: Target heart rate = (220 - age) × 60%-80% | — | 16weeks | 5 times a week | 90min | BW, BMI,TG, TC |
| Liu Wenjie,2013 | 42/41 | 56±1.35/56±1.35 | Square dancing/Tai chi | Personalized square dance training: square dances are self - organized collective square dances | Moderate to moderate-high intensity: 60%-80% of maximum heart rate | Intermittent monitoring: Control exercise load intensity through regular pulse tests, target intensity of 60% - 80% HRmax | 27weeks | 4 times a week | 60min | BW, BMI, RHR, SBP,VC |
| Joseph Keawe’aimoku  Kaholokula,2017 | 27/28 | 55±10/55±12 | Square dancing/Daily activities | Traditional culture square dance training: group practice of traditional dances and specific controlled rhythm movements | Moderate to high intensity: 40%-85% VO2Max (maximum oxygen uptake) or 50%-70% MPHR (maximum predicted heart rate) | Combined monitoring: 50% - 70% MPHR+ Borg Scale (Level 12 - 16) | 12weeks | 2 times a week | 60min | SBP |

Table note：BW = Body Weight; BMI = Body Mass Index; BF% = Body Fat Percentage; RHR = Resting Heart Rate; SBP = Systolic Blood Pressure; VC = Vital Capacity; TG = Triglycerides; TC = Total Cholesterol; HRmax = Maximum Heart Rate; VO₂max = Maximum Oxygen Uptake; MPHR = Maximum Predicted Heart Rate; RPE = Rating of Perceived Exertion


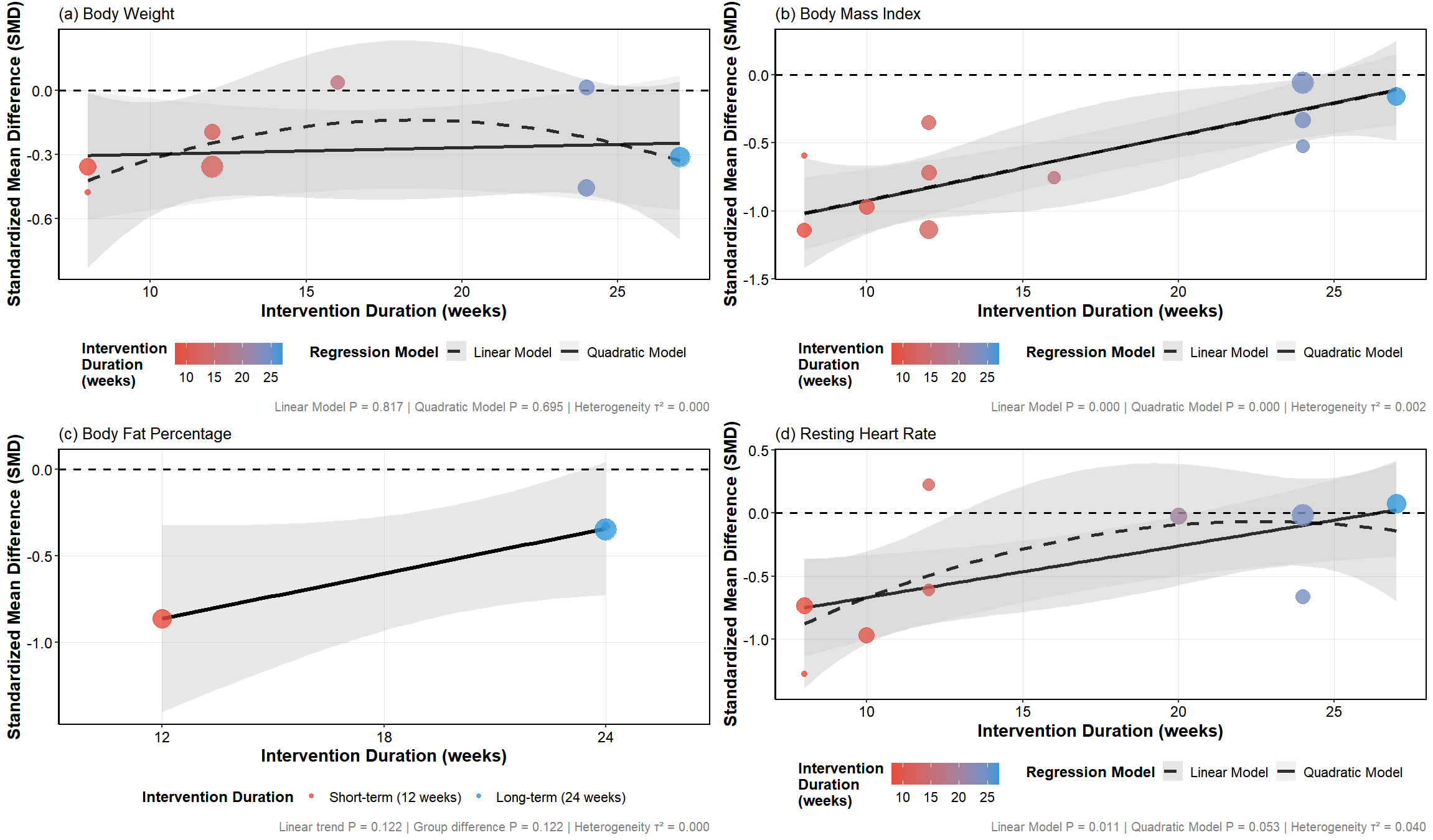


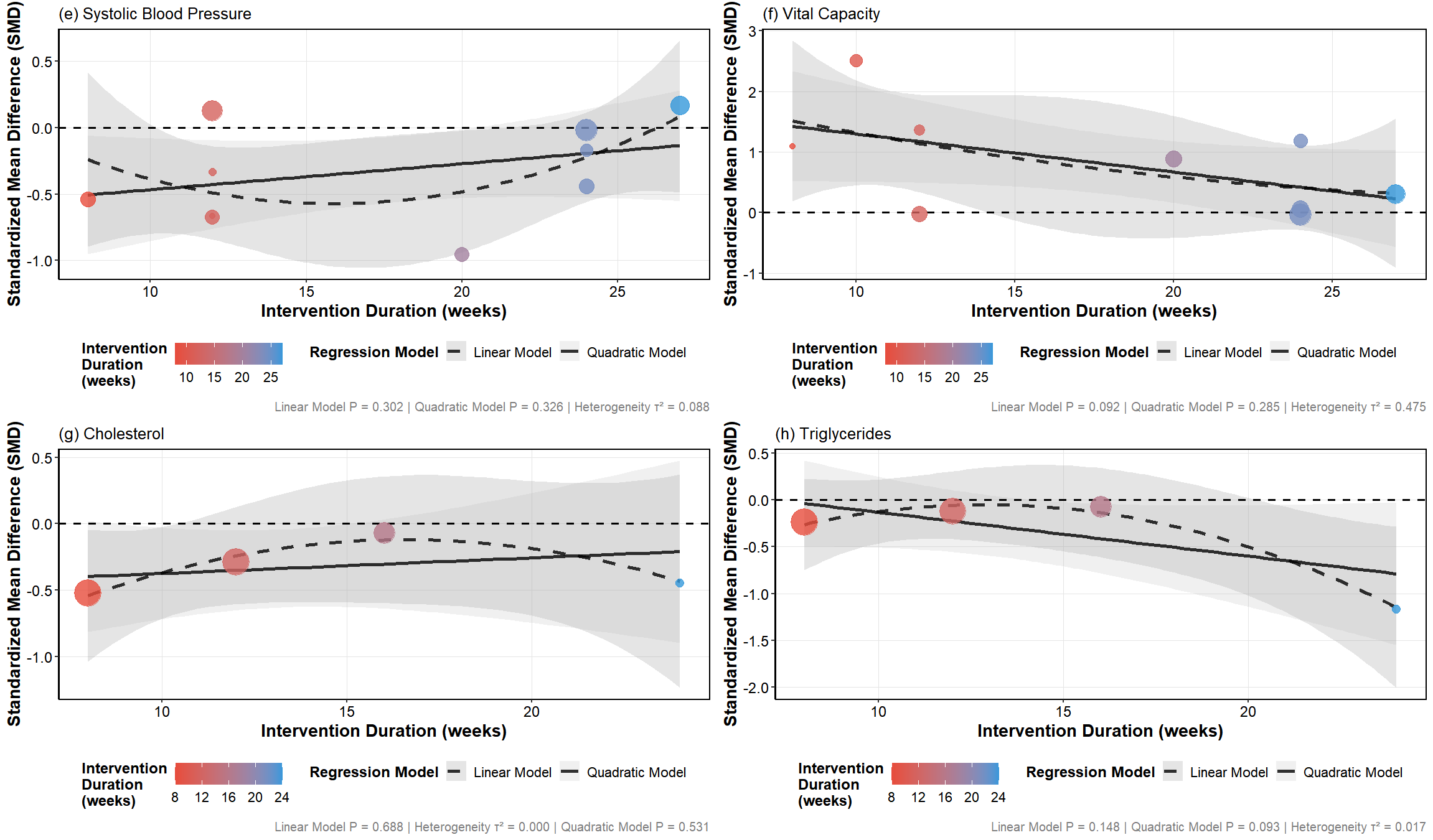


**Supplementary Figure 1.** Meta-regression analysis of the association between intervention duration and treatment effects
